# Supplementary material for: Spin current generation in organic antiferromagnets
Source: Nat Commun. 2019 Sep 20;10:4305. doi: 10.1038/s41467-019-12229-y (PMC6754401; doi:10.1038/s41467-019-12229-y)
Supplement: Supplementary file 1 — Supplementary Information [file 41467_2019_12229_MOESM1_ESM.pdf]

Supplementary Information:
Spin current generation in organic antiferromagnets

Naka *et al.*

(Dated: July 17, 2019)

Supplementary Note 1. Damping factor dependence of the spin current conductivity.

Supplementary Fig. 1a shows the damping factor η dependences of the spin current conductivity with respect to the thermal gradient, $\chi_{xy}^{\rm SQ}$, for the several values of the next-nearest-neighbor (NNN) exchange interaction K at $k_{\rm B}T = 0.5$ meV, obtained by the spin wave approximation. The exchange interactions are chosen as $(J, J', K') = (80, 20, 0)$ meV. Meanwhile, Supplementary Fig. 1b is the damping factor γ dependences of the spin current conductivity with respect to the electric field, $\chi_{xy}^{\rm SC}$, for the several values of the number of electrons in the unit cell n at $U = 1$ eV, obtained by the mean field theory. The transfer integrals are $(t_{\rm a}, t_{\rm p}, t_q, t_{\rm b}) = (-0.207, -0.102, 0.043, -0.067)$ eV. We note that both $\chi_{xy}^{\rm SQ}$ and $\chi_{xy}^{\rm SC}$ are inversely proportional to the damping factors and diverge in the clean limits ($\eta = 0$ and $\gamma = 0$). The sign change of $\chi_{xy}^{\rm SC}$ between $n = 6.2$ and 6.4 is associated with the change in the Fermi surface topology (Lifshitz transition); see the insets of Fig. 5a of the main text.

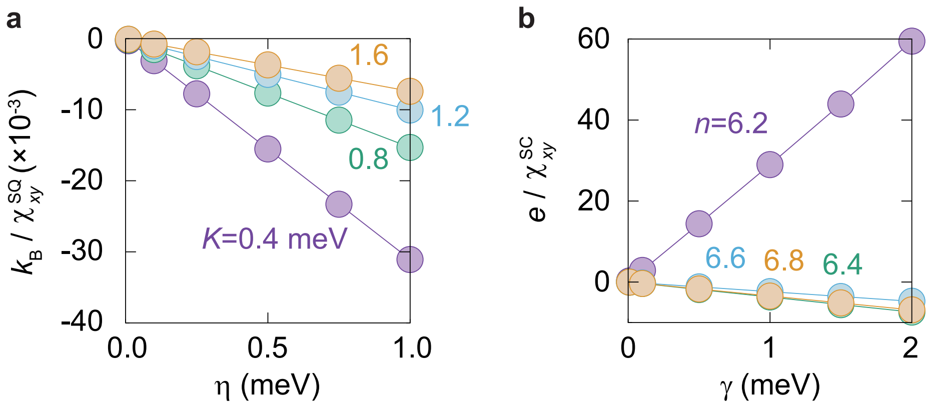

Supplementary Fig. 1. **Damping factor dependences of the spin current conductivities.** **a,** The damping factor η dependences of the inverse of the spin current conductivity, along the x-axis with respect to the thermal gradient along the y-axis, $1/\chi_{xy}^{\rm SQ}$, for the several values of K at $k_{\rm B}T = 0.5$ meV. The exchange interactions are fixed at $(J, J', K') = (80, 20, 0)$ meV. **b,** The damping factor γ dependences of the inverse of the spin current conductivity, along the x-axis with respect to the electric field along the y-axis, $1/\chi_{xy}^{\rm SC}$, for the several values of n at $U = 1$ eV. The transfer integrals are chosen as $(t_a, t_p, t_q, t_b) = (-0.207, -0.102, 0.043, -0.067)$ eV.

Supplementary Note 2. Derivation of the next-nearest-neighbor exchange interactions.

We derive the exchange interactions resulting in the spin splitting of the magnon dispersion in the antiferromagnetic insulating state. As explained in the main text, the difference of the real-space anisotropy between the A and B dimers reflecting the molecular orientations is incorporated in the electron transfer integrals on the NNN bonds. They arise from the second-order perturbation processes with respect to the nearest-neighbor (NN) transfer integral $\tau_{ij}^{\nu\bar{\nu}}$ in Eq. (3) of the main text. As the lowest-order exchange interactions reflecting the molecular orientations arise from the second-order perturbation in terms of the NNN transfer, they are given by the fourth-order perturbation processes in terms of the NN inter-dimer transfer.

We divide the Hubbard model for κ-type BEDT-TTF system [Eq. (1) of the main text] into two terms as

$$\mathcal{H}_{\rm Hubb} = \mathcal{H}^{(0)} + \mathcal{V}. \tag{1}$$

Hereafter, we take the hole picture instead of electron, which is convenient for the perturbation calculations. The first term describes local electronic states of isolated dimers, which is given by

$$\mathcal{H}^{(0)} = U \sum_{i\mu} n_{i\mu\uparrow}^{h} n_{i\mu\downarrow}^{h} - t_a \sum_{i\sigma} (h_{ia\sigma}^{\dagger} h_{ib\sigma} + \text{H.c.}), \tag{2}$$

where $h_{i\mu\sigma}$ and $n_{i\mu\sigma}^{h} (= h_{i\mu\sigma}^{\dagger} h_{i\mu\sigma})$ are the annihilation operator and the number operator of a hole with a spin $\sigma (=\uparrow, \downarrow)$, on the frontier orbital of molecular site $\mu (= a, b)$ in the ith dimer, respectively, U is the intra-molecular Coulomb interaction, and t_a is the intra-dimer transfer integral. The second term in Supplementary Eq. (1) represents the inter-dimer electron transfers, which is given by

$$\mathcal{V} = - \sum_{\langle ij \rangle \mu\mu'\sigma} t_{ij}^{\mu\mu'} (h_{i\mu\sigma}^{\dagger} h_{j\mu'\sigma} + \text{H.c.}), \tag{3}$$

where $t_{ij}^{\mu\mu'}$ is the transfer integral between the μ molecule in the ith dimer and μ' molecule in the jth dimer. The network of transfer integrals is described in Fig. 2b of the main text.

We first introduce the eigenstates of an isolated dimer described by $\mathcal{H}^{(0)}$. At three-quarter filling, where one hole occupies a dimer, the eigenstates are given by the bonding

and antibonding states: $|\beta_\sigma\rangle = (|a_\sigma\rangle + |b_\sigma\rangle)/\sqrt{2}$ with the energy $E_\beta = -t_a$ and $|\alpha_\sigma\rangle = (|a_\sigma\rangle - |b_\sigma\rangle)/\sqrt{2}$ with the energy $E_\alpha = t_a$. In the case where two holes exist on a dimer, the eigenstates are given by the following six states: the spin triplet states $\{|T_\uparrow\rangle, |T_0\rangle, |T_\downarrow\rangle\} = \{|\alpha_\uparrow\beta_\uparrow\rangle, (|\alpha_\uparrow\beta_\downarrow\rangle + |\alpha_\downarrow\beta_\uparrow\rangle)/\sqrt{2}, |\alpha_\downarrow\beta_\downarrow\rangle\}$ with the energy $E_T = 0$, the singlet state $|S\rangle = (|\alpha_\uparrow\beta_\downarrow\rangle - |\alpha_\downarrow\beta_\uparrow\rangle)/\sqrt{2}$ with the energy $E_S = U$, and the doubly-occupied states $|D^\pm\rangle = D^\pm_\alpha|\alpha_\uparrow\alpha_\downarrow\rangle + D^\pm_\beta|\beta_\uparrow\beta_\downarrow\rangle$ with the energies $E^\pm_D = U/2 \pm \sqrt{4t_a^2 + U^2/4}$. The coefficients are given by $D^\pm_\alpha = (U - E^\pm_D - 2t_a)/\sqrt{2(E^\pm_D - U)^2 + 8t_a^2}$ and $D^\pm_\beta = (E^\pm_D - U - 2t_a)/\sqrt{2(E^\pm_D - U)^2 + 8t_a^2}$. By using these α and β orbitals, the inter-dimer electron transfer term in Supplementary Eq. (3) is transformed as

$$\mathcal{V} = -\sum_{\langle ij\rangle\nu\nu'\sigma} \tau^{\nu\nu'}_{ij} (\tilde{h}^\dagger_{i\nu\sigma}\tilde{h}_{j\nu'\sigma} + \text{H.c.}), \tag{4}$$

where $\tilde{h}_{i\nu\sigma}$ is the annihilation operator of a hole in $\nu(=\alpha,\beta)$ orbital in the ith dimer. The transfer integral is given by the 2×2 matrix as

$$\tau_{ij} = -\frac{1}{2}\begin{pmatrix} t^{aa}_{ij} - t^{ab}_{ij} - t^{ba}_{ij} + t^{bb}_{ij} & t^{aa}_{ij} + t^{ab}_{ij} - t^{ba}_{ij} - t^{bb}_{ij} \\ t^{aa}_{ij} - t^{ab}_{ij} + t^{ba}_{ij} - t^{bb}_{ij} & t^{aa}_{ij} + t^{ab}_{ij} + t^{ba}_{ij} + t^{bb}_{ij} \end{pmatrix}. \tag{5}$$

Next we treat $\mathcal{V}$ as the perturbation term and calculate the exchange interactions in the fourth order in the restricted subspace where each antibonding orbital is occupied by one hole. The general form of the fourth-order perturbation process is given by Eq. (6) of the main text.

The real-space anisotropy in the electron transfer requires the second order of the inter-orbital transfer integrals, i.e., the off-diagonal elements of τ_{ij}, which depends on the relative phases between the bonding and antibonding orbitals, as shown in Supplementary Fig. 2. Then, the fourth-order perturbation processes, which bring about the anisotropic NNN exchange interactions, are classified into two types: one is the orders of $(\tau^{\nu\bar{\nu}})^4$ (type I) and the other is of $(\tau^{\nu\bar{\nu}})^2(\tau^{\nu\nu})^2$ (type II) with $\bar{\nu} = \beta$ (α) for $\nu = \alpha$ (β). Supplementary Figs. 3a-3c show the examples of the type-I and type-II perturbation processes on the NNN bond between the ith and kth dimers through the jth dimer, denoted by ijk. In the type-I process shown in Supplementary Fig. 3a, the hole on the α orbital in the ith dimer transfers to the α orbital in kth dimer through the β orbital in the jth dimer, by the second-order process of the off-diagonal elements of τ_{ij} and τ_{jk}, and goes back through the same pathway. On the other hand, in the type-II process shown in Supplementary Fig. 3b, the forward pathway

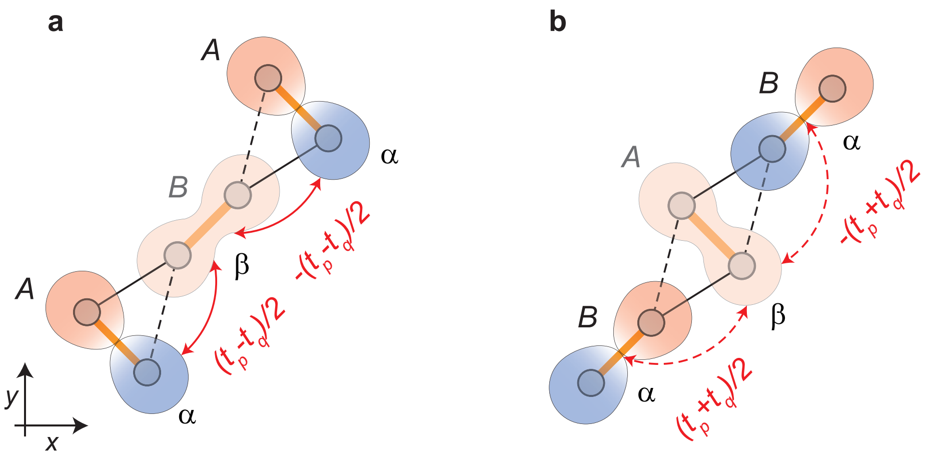

Supplementary Fig. 2. **Schematic illustrations of the inter-orbital NN electron transfer integrals.** **a,b,** The inter-orbital NN transfer integrals between the antibonding (α) orbital and bonding (β) orbitals leading to the real-space anisotropy of the NNN transfer integrals on A-B-A bond (**a**) and B-A-B bond (**b**). The orange and blue shaded ovals represent the positive and negative phases of the wave functions, respectively.

is the same with the type I, while the backward one is different and given by the diagonal elements of τ_{ij} and τ_{jk}. The type-II process has another pathway starting from the transfer of the hole on the jth dimer as shown in Supplementary Fig. 3c. Both the type-I and type-II processes give the Heisenberg-type exchange interaction in Eq. (7) of the main text.

Let us explicitly show the forms of the coupling constants $\tilde{J}$ and K, separately for the type-I and type-II processes as $\tilde{J} = J_{\rm I} + J_{\rm II}$ and $K = K_{\rm I} + K_{\rm II}$. The explicit forms are given by

$$\tilde{J}_{\rm I} = (\tau_{ij}^{\alpha\beta}\tau_{jk}^{\beta\alpha})^2 \left(\frac{D_\beta^+ D_\alpha^-}{E_D^- - 2t_a} - \frac{D_\beta^- D_\alpha^+}{E_D^+ - 2t_a} \right) \left(\frac{1}{E_T - 2t_a} - \frac{1}{E_S - 2t_a} \right)^2, \tag{6}$$

$$K_{\rm I} = (\tau_{ij}^{\alpha\beta}\tau_{jk}^{\beta\alpha})^2 \left(\frac{D_\beta^+ D_\alpha^-}{E_D^- - 2t_a} - \frac{D_\beta^- D_\alpha^+}{E_D^+ - 2t_a} \right) \frac{4}{E_T - 2t_a} \left(\frac{1}{E_T - 2t_a} + \frac{1}{E_S - 2t_a} \right), \tag{7}$$

$$\tilde{J}_{\rm II} = \tau_{ij}^{\alpha\beta}\tau_{jk}^{\beta\alpha}\tau_{ij}^{\alpha\alpha}\tau_{jk}^{\alpha\alpha} \left(\frac{D_\beta^+ D_\alpha^-}{E_D^- - 2t_a} - \frac{D_\beta^- D_\alpha^+}{E_D^+ - 2t_a} \right) \left[2 \left(\frac{1}{E_T - 2t_a} - \frac{1}{E_S - 2t_a} \right) - \frac{1}{t_a} \right], \tag{8}$$

$$K_{\rm II} = \tau_{ij}^{\alpha\beta}\tau_{jk}^{\beta\alpha}\tau_{ij}^{\alpha\alpha}\tau_{jk}^{\alpha\alpha} \left(\frac{D_\beta^+ D_\alpha^-}{\lambda^- - 2t_a} - \frac{D_\beta^- D_\alpha^+}{\lambda^+ - 2t_a} \right) \left(\frac{4}{V_a - 2t_a} + \frac{1}{t_a} \right). \tag{9}$$

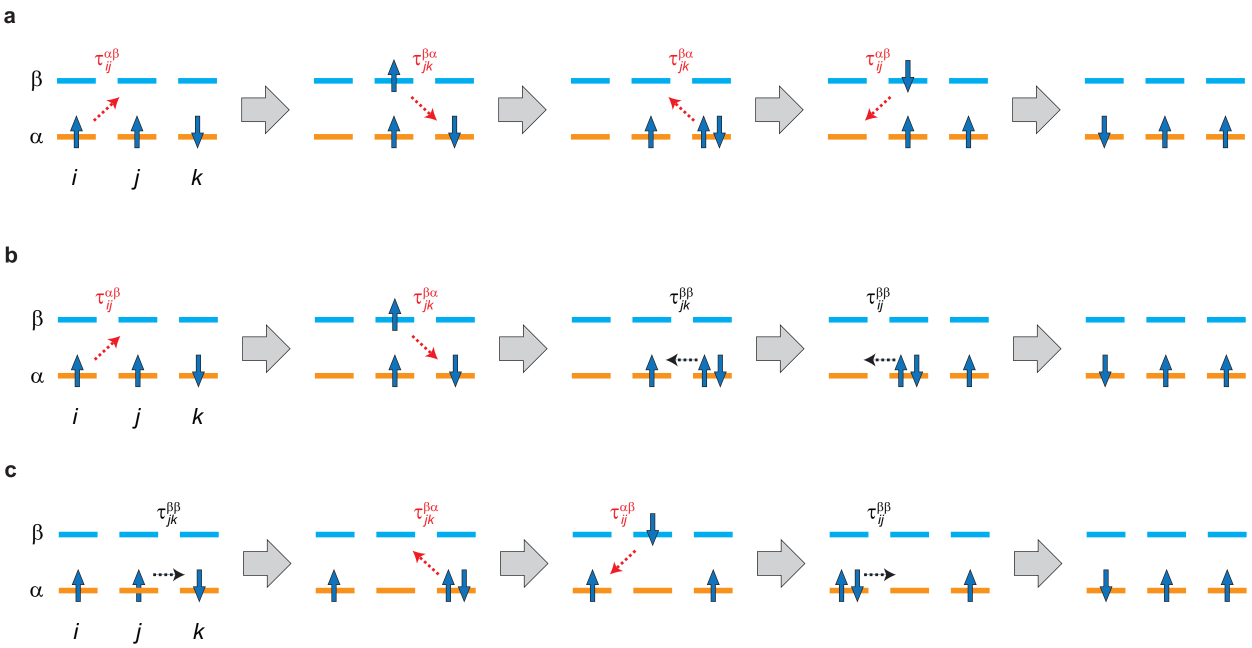

Supplementary Fig. 3. **Schematic illustrations of the NNN exchange processes in the hole picture. a,** A type-I perturbation process of the order of $(\tau^{\nu\bar{\nu}})^4$. The orange and blue solid lines represent the energy levels of the antibonding (α) orbital and bonding (β) orbital, respectively. The red (black) broken arrows are the inter-orbital (intra-orbital) transfer integrals. **b,c,** Type-II perturbation processes of the order of $(\tau^{\nu\bar{\nu}})^2(\tau^{\nu\nu})^2$, starting from the left dimer (**b**) and the mid dimer (**c**).

The amplitudes of $\tilde{J}$ and K depend on the molecular orientations in the NNN bonds shown in Figs. 4a in the main text. We define them as $(\tilde{J}, K)$ for A-B-A bond and $(\tilde{J}', K')$ for B-A-B bond. The difference between K and K' results in the spin splitting of the magnon band shown in Fig. 4c in the main text. On the other hand, the difference between $\tilde{J}$ and $\tilde{J}'$ does not contribute to the spin splitting, because the NN exchange interaction for every A-B bond is given by $\tilde{J} + \tilde{J}'$, which does not show the real-space anisotropy. Then, they can be absorbed in the definition of J, and since the amplitude of $\tilde{J} + \tilde{J}'$ is much smaller than the second-order NN exchange interaction J, we neglect $\tilde{J}$ and $\tilde{J}'$ in the present calculation. The other fourth-order exchange processes on ring-type paths are also not taken into account. This is because, as shown in Figs. 3a and 3b of the main text, the spatial anisotropy of the second-order electron transfers reflecting the molecular orientation appears only on the NNN bonds in the $\langle 11 \rangle$ direction, which do not contribute to the ring-type exchange processes.
